# A Rare Case of an Isolated Neglected Subcutaneous Rupture of the Flexor Digitorum Superficialis in a Climber: Successful Surgical Treatment and Review of the Literature

**DOI:** 10.7759/cureus.36055

**Published:** 2023-03-12

**Authors:** Walid Bouziane, Ziyed Missaoui

**Affiliations:** 1 Department of Orthopedic Surgery and Traumatology, University Hospital Mohamed VI Oujda, Oujda, MAR; 2 Department of Orthopedic Surgery, Grand Hopital de I'Est Francilien, Meaux, FRA

**Keywords:** rare case report, climber, hand surgery, flexor digitorum superficialis, subcutaneous rupture

## Abstract

Isolated neglected subcutaneous rupture of the flexor digitorum superficialis (FDS) in zone III of the hand is a rare injury that is distinct from subcutaneous rupture of the deep flexor tendons in the fingers. While a few cases have been reported in the literature, the pathophysiology of this injury remains poorly understood.

In this article, we present a case study of a climber who experienced an isolated subcutaneous rupture of the FDS following a sports accident. The patient's diagnosis was initially delayed due to an unclear clinical presentation. However, surgical intervention was successfully performed, resulting in positive clinical outcomes at the one-month follow-up. This case highlights the importance of considering isolated subcutaneous rupture of the FDS as a potential injury in climbers and other individuals who engage in high-impact sports.

## Introduction

Although isolated neglected subcutaneous rupture of the flexor digitorum superficialis (FDS) is often considered a rare injury, it should not be disregarded [1]. While a limited number of cases have been reported in the medical literature, it is recognized that ruptures can occur as a result of a systemic condition like rheumatoid arthritis or localized issues such as malunion, Kienbock's disease, or pseudarthrosis of the capitate or hamate [2]. The pathophysiology of these conditions is well-established. Diagnosis is based on clinical evaluation, and confirmation can be obtained via ultrasound or magnetic resonance imaging (MRI). Treatment options are influenced by the chronicity of the injury [2].

In this article, we present a case study of a climber who experienced an isolated subcutaneous rupture of the FDS in zone III of the hand. The patient was treated surgically and had a favorable outcome.

## Case presentation

**Figure 1:**
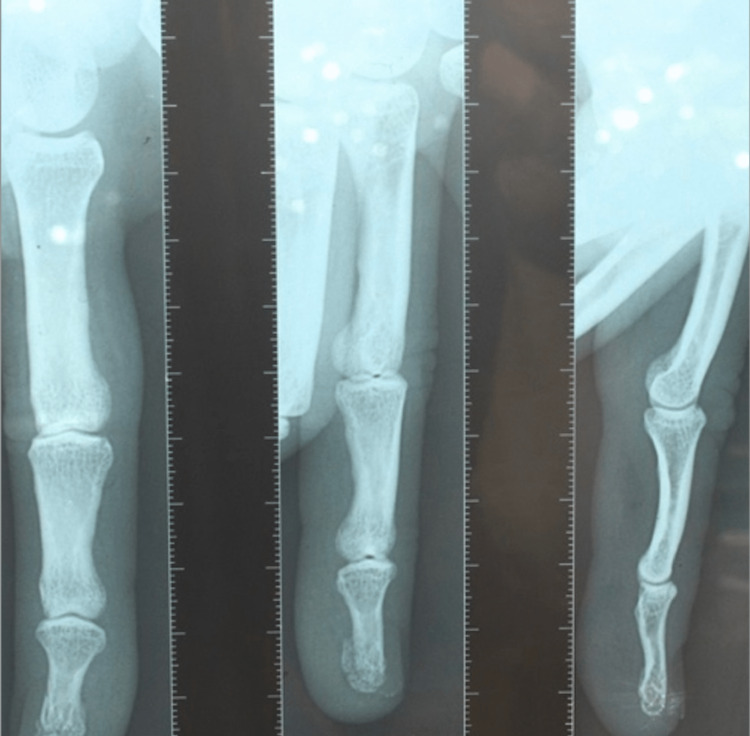
Standard radiographs of the 3rd finger, anteroposterior and lateral views, without anomalies

We present the case of a 38-year-old right hand dominant climber with no comorbidities who visited the emergency department after experiencing a cracking sensation in the third finger during a training session. Standard radiography did not reveal any abnormalities (Figure 1). A preliminary diagnosis of high-grade proximal interphalangeal joint sprain was made, and the patient was treated with orthopedic hand bracing and analgesics.

After three weeks, the patient had no improvement and continued to experience pain and limited range of motion in the affected finger. An ultrasound was performed, which did not reveal any tendon rupture. Conservative treatment was continued with physiotherapy.

Two months later, the patient presented to our hospital with persistent discomfort during the flexion of the third finger and a nodule in the third zone of the flexors in the left hand. Clinical examination showed complete but difficult flexion of the finger. A comparative hand examination revealed a deficit in flexion of the proximal interphalangeal joint compared to the healthy side.

**Figure 2:**
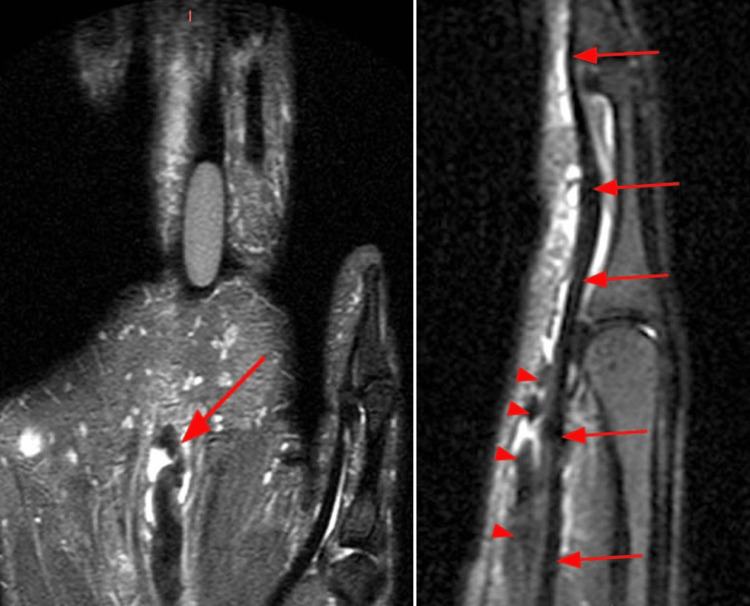
MRI images of the left hand MRI images clearly demonstrate the intact FDP tendon (arrows) found deep to the proximally retracted and redundant FDS, compatible with an isolated closed rupture. FDP: Flexor digitorum profundus, FDS: Flexor digitorum superficialis

An MRI confirmed an isolated rupture of the third tendon of the FDS in the left hand, which had retracted to zone III. The flexor digitorum profundus tendon was found to be intact (Figure 2), and surgical treatment was recommended.

**Figure 3:**
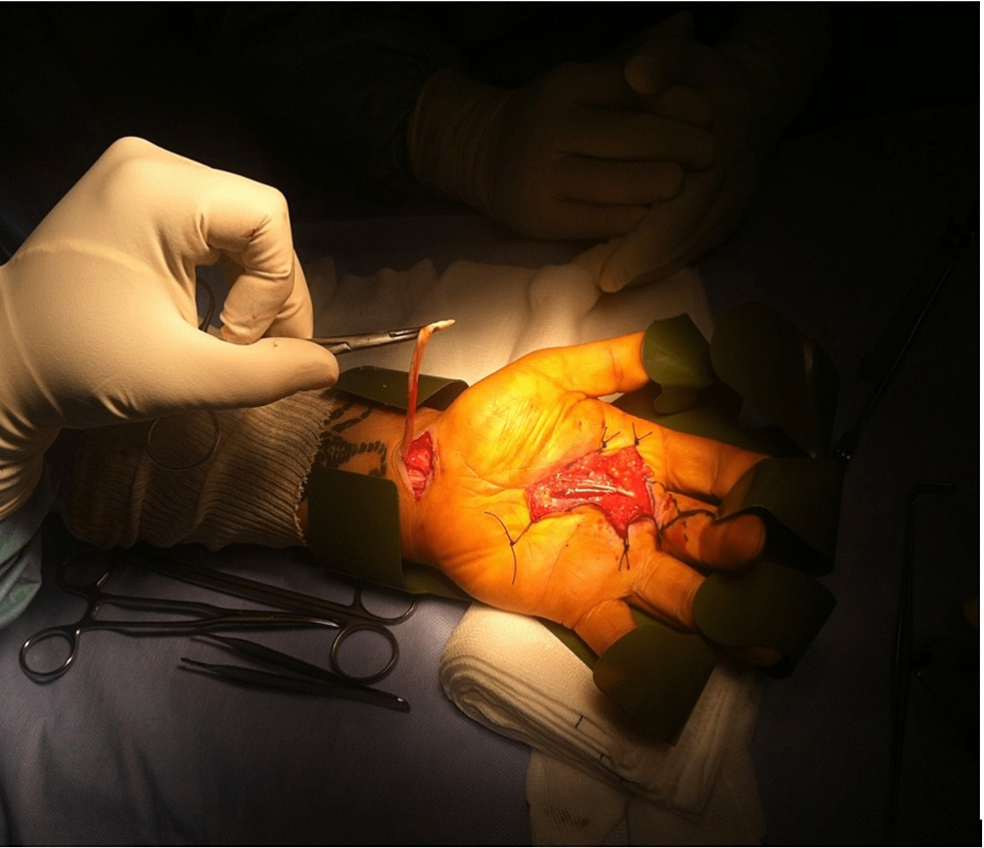
Intraoperative image The image shows the ruptured superficial flexor tendon of the left 3rd finger found in zone III.

Under locoregional anesthesia, a Z-shaped incision was made over the nodule. The stump of the ruptured tendon was found in zone III , and it was resected due to its poor quality and retraction. The flexor digitorum profundus was embedded in fibrotic scar tissue, so tenolysis was performed and pulleys were intact (Figure 3).

The postoperative course was simple, no immobilization was done, a self-rehabilitation was started on day 2 post-operation after the subsidence of the pain.

**Figure 4:**
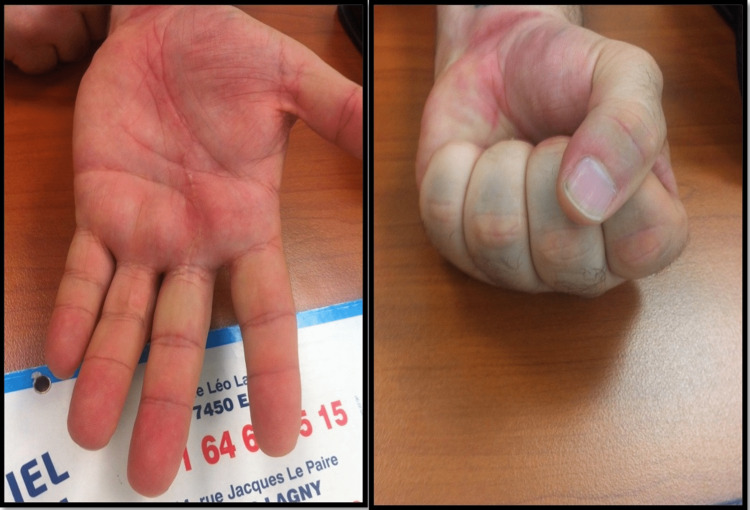
Clinical results after one month

The patient was followed up for two years. No skin complications were reported (Figure 4), and the QuickDASH score [3] was 6/100 at one month. The patient was authorized to resume sporting activities from the fourth week, and there were no sequelae.

## Discussion

Non-pathological subcutaneous rupture of the FDS of the fingers is a rare diagnosis in current hand surgery practice, and few cases have been published in the literature. In 2007, Bois et al. [1] reviewed 50 years (1956-2006) of literature, describing 50 cases of spontaneous flexor rupture, including six cases of isolated FDS rupture which were always associated with a rupture of the FDP. The mechanism of injury is usually due to forceful contraction of the FDS tendon against a fixed hyperextended proximal interphalangeal joint. Other mechanism of injury includes the non-traumatic ruptures secondary to an inflammatory pathology such as rheumatoid arthritis or, to a lesser degree, tenosynovitis or tuberculosis is well known [2]. 

Several hypotheses have been proposed to explain the rarity of non-pathological ruptures of the FDS at the fingers. One such hypothesis is that the footprint of the FDP on the distal phalanx is narrower than that of the FDS on the middle phalanx, thus making the FDP more important in finger flexion and more susceptible to injury [2]. Folmar et al. [4] described this lesion in two patients, while Boyes et al. [5] described it in three patients, and both authors recommended non-surgical treatment. Stern et al. [6] published a series of 11 cases in 1995, noting that the ring finger was the most frequently involved and that time to diagnosis had an inverse relationship with functional results after surgery. Ferraro and Schenck [7] reported a surgically treated avulsion of the bony insertion of the FDS tendon. Vandeputte and Dubert [8] reported a surgically treated closed avulsion of the FDS with avulsion of the annular pulleys. In general, zone III flexor tendon injuries are relatively rare in comparison to other flexor tendon injuries in zones I, II, IV, and V. Often, these are open injuries resulting from an electrical device like a saw; however, closed injures are even rarer [9]. 

The clinical presentation is almost always the same: deformation of the proximal inter phalangeal joint in flexion, persistent pain in the hand and reduced range of motion of the proximal inter phalangeal joint [10]. In most cases, patients describe a minor trauma that is often neglected, which can lead to delayed diagnosis [10]. The unclear clinical picture and difficult examination during the acute phase, along with the compensatory actions of the FDP, can make initial diagnosis challenging. However, a meticulous and comparative examination of both hands can often help to clarify the diagnosis.

The role of standard radiography in the diagnosis of non-pathological ruptures of FDS of the fingers is to detect a bone avulsion at the FDS insertion and assess the condition of the interphalangeal joint. Ultrasound is useful for confirming the diagnosis, quantifying retraction, and identifying the location of the rupture, which is often at the level of the A1 pulley or in Camper's Chiasm. It can also be used to assess for associated pulley or FDP lesions. While MRI is not always necessary, a well-performed ultrasound by a trained radiologist can provide the information needed for surgical planning.

An anatomical classification of this lesion was proposed by Alsaadi et al. [2] based on previous scientific research. The classification includes five types: closed avulsion of one or both slips of the FDS, closed rupture with intact stump, closed avulsion of both slips of the FDS with annular pulley, closed avulsion of the bony insertion of the FDS, and closed avulsion of both the FDS and the FDP.

The surgical technique proposed by Stern et al. [6] involves a palmar zigzag incision starting at the distal interphalangeal joint and extending into the palm, with the retracted ends of the superficial tendon located at the level of the A1 pulley. In addition to excising scar tissue, joint release and capsulotomy may be necessary in cases of stiffness.

Caso et al. [11] reported an atypical case of FDS tendon rupture in a male climber who used a specific sport climbing grip known as the "hook grip," characterized by extension of the metacarpophalangeal joints and maximum flexion of the proximal interphalangeal joints with forces applied only to the middle phalanx of the middle finger. This case was treated non-operatively and achieved good long-term results after physiotherapy.

Our case is an isolated non-pathological FDS tear with delayed diagnosed. We believe this lesion is underestimated, and its incidence is greater than believed, so we insist that each patient must undergo a comparative clinical examination of both hands irrespective of their pathology. The diagnosis is generally clinical, and the treatment is classical, including the release and excision of the cicatricial fibrosis. No postoperative immobilization is necessary thus a self-rehabilitation of the fingers must begin immediately.

## Conclusions

Although isolated neglected subcutaneous rupture of the FDS is considered rare, we believe that its incidence may be greater than believed. We believe that a careful, bilateral, and comparative clinical examination in the face of even minimal hand trauma and prompt surgery can prevent prolonged and potentially irreversible disability.
